# Managing of Unassigned Mass Spectrometric Data by Neural Network for Cancer Phenotypes Classification

**DOI:** 10.3390/jpm11121288

**Published:** 2021-12-03

**Authors:** Denis V. Petrovsky, Arthur T. Kopylov, Vladimir R. Rudnev, Alexander A. Stepanov, Liudmila I. Kulikova, Kristina A. Malsagova, Anna L. Kaysheva

**Affiliations:** 1 Biobanking Group, Branch of Institute of Biomedical Chemistry “Scientific and Education Center”, 109028 Moscow, Russia; petro2017@mail.ru (D.V.P.); a.t.kopylov@gmail.com (A.T.K.); v.r.rudnev@gmail.com (V.R.R.); aleks.a.stepanov@gmail.com (A.A.S.); likulikova@mail.ru (L.I.K.); kaysheva1@gmail.com (A.L.K.); 2 Institute of Theoretical and Experimental Biophysics, Russian Academy of Sciences, 142290 Moscow, Russia

**Keywords:** cancer, neural network, system biology, bioinformatics, proteomics, metabolomics, multiomics data

## Abstract

Mass spectrometric profiling provides information on the protein and metabolic composition of biological samples. However, the weak efficiency of computational algorithms in correlating tandem spectra to molecular components (proteins and metabolites) dramatically limits the use of “omics” profiling for the classification of nosologies. The development of machine learning methods for the intelligent analysis of raw mass spectrometric (HPLC-MS/MS) measurements without involving the stages of preprocessing and data identification seems promising. In our study, we tested the application of neural networks of two types, a 1D residual convolutional neural network (CNN) and a 3D CNN, for the classification of three cancers by analyzing metabolomic-proteomic HPLC-MS/MS data. In this work, we showed that both neural networks could classify the phenotypes of gender-mixed oncology, kidney cancer, gender-specific oncology, ovarian cancer, and the phenotype of a healthy person by analyzing ‘omics’ data in ‘mgf’ data format. The created models effectively recognized oncopathologies with a model accuracy of 0.95. Information was obtained on the remoteness of the studied phenotypes. The closest in the experiment were ovarian cancer, kidney cancer, and prostate cancer/kidney cancer. In contrast, the healthy phenotype was the most distant from cancer phenotypes and ovarian and prostate cancers. The neural network makes it possible to not only classify the studied phenotypes, but also to determine their similarity (distance matrix), thus overcoming algorithmic barriers in identifying HPLC-MS/MS spectra. Neural networks are versatile and can be applied to standard experimental data formats obtained using different analytical platforms.

## 1. Introduction

According to the central dogma of molecular biology proposed by Francis Crick (1958), the information genetically encoded in DNA is transformed to proteins, and subsequently through enzyme-mediated transformation into to metabolites, via RNA. The result of this genetically determined transition is the synthesis of biological molecules, proteins, and metabolites that implement a genetically programmed trait or phenotype [1]. Therefore, by observing the expression of a certain set of genes in an experiment, a researcher expects to confirm the information encoded by these genes at the subsequent molecular levels—transcriptomic, proteomic, and metabolomic.

At the end of the 20th century, studies began to appear in the literature indicating a weak correlation between the components of different molecular layers, primarily between the expression levels of mRNA and protein in biological samples [2]. Today, it is well known that the general genome correlation between the level of expression of mRNA and proteins is only 40% [3,4,5]. Similar results were observed in a more complex correlation analysis between gene expression and metabolite formation [6]. The impressive discrepancy between the composition and the amounts of molecular components inhabiting different “omics layers” (mRNA, proteins, metabolites) is usually explained by the complex regulation of the processes of transcription and translation [7,8], as well as technological and algorithmic limitations of post-genomic analysis tools [9]. Researchers agree that a comprehensive understanding of the state of the human body in both the healthy state and disease requires the integration of knowledge about the qualitative and quantitative content of molecular components in a biological sample from several molecular levels, such as the genome, epigenome, transcript, proteome, and metabolome [10,11,12]. The rapid development of high-throughput technology for sequencing and mass spectrometric analysis of molecular profiling has led to a high rate of generation of “multiomics” data and has defined the era of “big data” in the study of biological processes. Today, the global problem of the practical application of the results of molecular profiling is multivariate data mining.

Oncological diseases are perhaps the biggest challenge to modern biomedicine, as these are pathologies with a high mortality and disability rate [13]. Cancer is a multigenic and, at the same time, multifactorial pathology and is accompanied by the dysregulation of a large number of genes [14]. The main result of this dysregulation is the reprogramming of a healthy phenotype cell into an oncological one [15]. The pathological processes accompanying the development of any oncological disease are similar in many respects [9]. Common signs of oncopathology include the maintenance of proliferative signaling, avoidance of growth suppressors, disruption of the mechanisms of cell apoptosis, provision of replicative immortality, induction of angiogenesis, activation of invasion and metastasis, and reprogramming of energy metabolism and evasion of immune destruction [14]. Due to the similarity of developmental mechanisms, the identification of serological markers (proteins and metabolites) of a separate oncopathology is difficult [9,16].

In this regard, the goal of this study was to create a neural network for the classification of patients with prostate cancer, ovarian cancer, and kidney cancer. We analyzed the mass spectrometric proteo-metabolomic study of patients with various types of cancer [9], of which two were classified as sex-specific. The data obtained were divided into three sets: training, validation, and testing, without prior identification.

Several popular machine learning (ML) tools utilize mass spectrometric (MS) data for the classification of phenotypes. The most widely employed for initial data processing are support-vector networks (SVN) [17], random forest (RF) [18], and linear discriminant analysis (LDA) [19]. Due to the lack of standardized recommendations, comparative analysis is challenging if operating with MS-based data. The mass spectrometry-based data pre-processing is essential for stabilization improvement of multidimensional data [20]. Depending on the quality of the pre-processed data and the model chosen, the adequacy of biological assumptions may vary widely. Therefore, the selection of descriptors and accounting for their variation are essential for consolidation of the outcome. Generally, this task encompasses extraction of descriptors through their local extremums. However, we applied an end-to-end approach that learns representations directly from the mass spectra data. A typical convolutional neural network (CNN) includes layers that fit invariant features of an input signal. Therefore, a high predictability measure is the main advantage of the CNN classification regarding the raw MS-based data with the contribution of a noise signal and baseline variation.

The application of the neural network (NN) approach in biomedical settings is becoming of growing interest, although NN still cannot be utilized as a diagnostic tool due to many limitations related to data availability and cost-expensive experimental validation.

There are many reports of successful NN application on small and on large datasets. For instance, a research group from School of Engineering (University of Warwick, Coventry, UK) has been designed a successful NN model for the evaluation of osteoarthritis severity depending on the bone size and gender (male and female). The designed model has been obtained using the very small initial dataset (only 35 samples) avalible for training [21]. The small dimensionality issue can be resolved by a special NN tool, such as augmentation, by way of stochastic variation of the input data, which is critical for the mass spectrometry-based data [22,23,24,25]. A deep convolutional neural network has been successfully applied to poorly stratified thyroid cancer. The authors used ultrasound images as an input information of more than 180,000 totally collected different images from more than 17,000 patients [26]. The final output on the validation test demonstrated a sensitivity up to 94% and showed improved performance compared to the skilled radiologist. A convolutional neural network has been applied to data-independent (DIA) mass spectrometry data for peptides sequencing [27]. The authors collected DIA spectra (a specially developed approach in mass spectrometry with no information loss unlike data-dependent scanning) and extracted the same features as we used in our study: *m*/*z*, intensity, and retention time. Typically, the DIA approach requires preliminary generation of a customized spectra library to be able to read and manage DIA data. However, this is a time-consuming and cost-expensive task. In this study, the authors used convolutional NN to couple DIA spectra with identifying novel peptides. However, the application is limited to antibodies and antigens due to sequence patterns predictability. NNs are becoming utilized to predict post-translational modification primarily due their flexibility and performance. Kinase-specific phosphorylation and general phosphorylation sites can be predicted by the MusiteDeep tool, which uses UniProt KB annotated phosphorylation sites as an input positive control and transfer general phosphorylation sited to train the model for the kinase-specific prediction [28]. The DeepPhispho tool employs intra- and inter-block concatenation layers architecture to generate phosphorylation predictions through capturing a multiple representation of sequences [29]. However, both neural networks are characterized by a limited predictability, which is caused by a limited number of sufficient substrates for known kinases (over 95% of known substrates do not have known upstream kinases [30]). Deep machine learning has been proposed to classify clinically relevant samples that were analyzed by the LC-SRM method, and the best classification AUC achieved 0.94 [31]. General disclination of normal (control) samples from the tumor ones can be achieved using a NN that operates with data-dependent (DDA) mass spectrometric data, and such an approach skips proteins and peptides identification typical for the traditional proteomic workflow [32].

In the present study for the analysis and classification of pathologies, two neural networks with the architecture of “residual convolutions” (1D residual CNN and 3D CNN) were developed, the input data for which were data of the mass spectrometric signal for proteins and metabolites. A distinctive feature of deep CNNs is their ability to receive data in its original form and transform it into feature maps. The trained neural network distinguishes three types of oncopathology with a high reliability, using metabolomic and proteomic spectra of patients’ blood plasma as an input.

The goal of this study is the building of a neural network algorithm capable of the efficient recognition of unrelated pathologies using both metabolomic and proteomic mass spectrometry-based data as complementary layers.

## 2. Materials and Methods

### 2.1. Subjects and Ethical Consideration

The study cohort comprised five groups of patients, each with any one of the following confirmed diagnoses and clinical records; ovarian cancer (OVC; *n* = 56; age 56.7 ± 9.1 years; stages Ic–IIIc), kidney cancer (RNC; *n* = 48, of them 34 males aged 56.7 ± 9.8 years, stages I–IV; and 14 females aged 57.8 ± 9.2 years, stages I–IV), and prostate cancer (PRC; *n* = 52; age 59.4 ± 5.3 years; stages II–III). All of the patients with cancer were enrolled from the Research Clinical Center for Oncology (Moscow, Russia). Patients with a previous history of chronic diseases were strictly excluded since the impact may significantly overlap with the cancer phenotype, making the differentiation almost impossible. The gender-specific cancer phenotype was considered against the corresponding gender-specific control subjects. The control group of healthy individuals comprised 40 subjects (20 males aged 61 ± 6.1 years and 20 females aged 50 ± 2.4 years). The study was approved by the independent local research ethics committee of the M.F. Vladimirsky Research Clinics (protocol no. 18 of 24 December 2020), Sechenov University (protocol no. 10-19 of 17 July 2019), and performed in accordance with the WMA Declaration of Helsinki on Ethical Principles for Medical Research Involving Human Subjects. All of the patients and healthy donors provided written informed consent to participate in the study.

### 2.2. Mass Spectrometry Data

Mass spectrometric data were obtained from the framework of the work [9]. The procedures for the preliminary preparation of samples and carrying out mass spectrometric measurements are described in detail in our previous study [9].

### 2.3. Data Analysis Using the Neural Network

After mass spectrometry detection, a total of 337 initial raw data files were converted into 300 standardized ‘mgf’ files (average size of a single datafile is 250 MB, the total size of the dataset is 240 GB). The mass spectrometric intensity and mass-to-charge ratios encoded in ‘mgf’ files were chosen as key descriptors aligned by the retention time (RT) with a 0.1-second step. The training dataset size comprised 60%, or 180 ‘mgf’ files, of the complete dataset, whereas the test dataset comprised 40%, or 120 ‘mgf’ files. Both the training and test datasets were composed of all of the collected pathologies and the control group in equal proportions. Discrimination of different stages within a specific cancer type was not carried out due to the small size of the study population.

Noise reduction of the extracted data was performed in the initial data handling and included the following steps: (a) extraction of retention time, intensity, and mass-to-charge features; (b) elimination of rare *m*/*z* features and intensities such that the frequency of each feature exceeded 2 in each dataset; (c) rounding each *m*/*z* feature to 10 ppm for proteomic data and 100 ppm for metabolomic data; (d) normalization of intensity and *m*/*z* features using the min-max scalar, and noise reduction using the elliptic envelope approach with an outlier fraction cut-off not exceeding 0.2, assuming that the weighted average error of mass spectrometric measurements is below 20%.

To control the performance and robustness of the designed model on small datasets, a data augmentation approach was applied to overcome data dimensionality. The initial dataset (337 ‘mgf’ files) was split into training (60%) fractions and testing and validation fractions (the remaining 40% of the total dataset). To prevent fraction fusion and mixing, the transformation was performed independently for each dataset. Up to 10% of the spectral data elements (5% from the starting section and 5% from the final section of the data files) were extracted from each ‘mgf’ file and recorded in the augmentation database to collect 10% of the total massive data into the augmentation database. Elements from the augmentation database were randomly picked up and incorporated into MS positions over the meaningful part of the data repeatedly for replicates to enlarge the initial dataset and split the resulting augmented dataset into training, testing, and validation (Table 1).

The converted data of the mass spectrometric signal were saved in the database (the document-oriented MongoDB was used as the database). As part of the work on data analysis using neural networks, two models were developed and tested with different options for presenting the initial data and different architectures.

**Figure 1:**
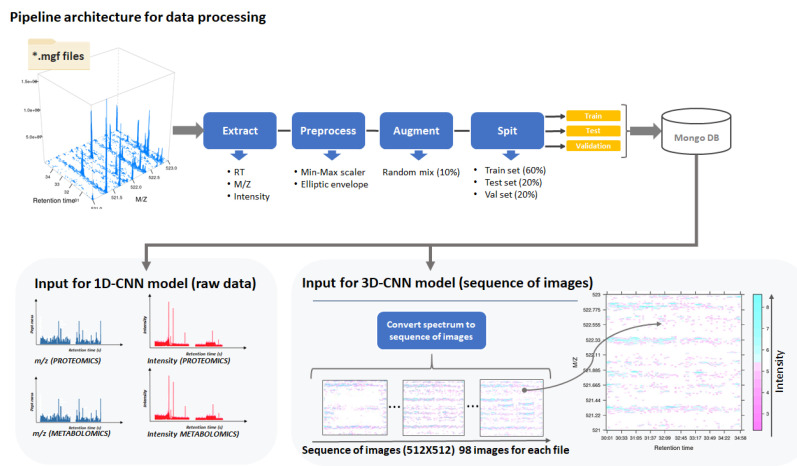
The schematic representation of 1D-CNN and 3D-CNN pipeline architecture for mass spectrometry-based data processing using data conversion into ‘mgf’ files.

The first model (1D-residual CNN) works with raw mass spectrometric signal data for proteins and metabolites. The input data for this model are presented in the form of four arrays: *m*/*z* and intensity for proteomic analysis, *m*/*z* and intensity for metabolic analysis. For the second model (3D-CNN), the MS signal is represented as a sequence of spectrum images, in which each image represents a portion of the signal spectrum with a duration of 96.7 s. The coordinates of each point are the values (retention time, *m*/*z*), and the signal intensity is color-coded. Thus, for each ‘mgf’ file from the original set, a sequence of 98 images with dimensions of 512 × 512 pixels was obtained. The total volume of images for proteins was 63,069 files, with a further 54,752 files for metabolites (Figure 1).

### 2.4. 1D-Residual CNN Model

**Figure 2:**
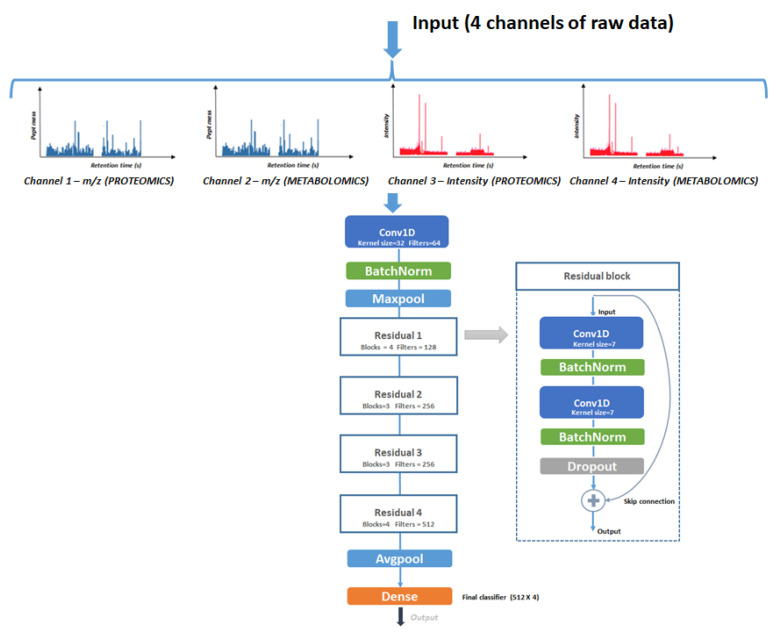
The schematic representation of 1D residual CNN architecture.

The first model is based on the dual CNN architecture and consists of 29 convolution layers with an output dense layer. The input data for the model are 4 channels: *m*/*z* and intensity of proteins and *m*/*z* and intensity of metabolites (Figure 2 and Table 2).

The proposed architecture had large receptive fields in the first convolutional layer because it was assumed that the first layers should have a more global view of the spectral signal. Moreover, the spectral signal is non-stationary, that is, the frequency or spectral content of the signal changes with respect to time. Therefore, shorter filters do not provide a general view of the spectral content of the signal. Residual blocks were implemented with double convolution layers that contained non-linear GELU activation Equation (1) and batch normalization in between to avoid the problem of vanishing/exploding gradient skip-connections between convolution layers. To reduce overfitting, dropout (*p* = 0.3) was applied.

(1) $$GELU\left(x\right)=xP\left(X\leq x\right)=x\Theta \left(x\right)≅0.5x\left(1+tanh\left(\sqrt{\frac{2}{\pi }}\left(x+0.044715x^{3}\right)\right)\right)$$

All of the convolutional layers in the residual blocks applied the same convolutional window of size 7 × 7, and the number of filters increased from 64 to 512 following the depth of the networks. To reduce the resolution of the input signal, a stride of two was applied after the first layer in the block.

The average pooling layer was applied at the end of all of the residual blocks. The output of the last average pooling was flattened and used as the input to the fully connected layer. As this is a classification task, the SoftMax function activation was applied to map neurons in the output layer in a range (0, 1) and sum them up to 1 to calculate the probability distribution for different pathologies (four classes: OVC, RNC, PRC, and the control). Multiclass cross-entropy was selected as a loss function. The Adaptive Moment Estimation (Adam) optimizer with a reduced learning rate was used when the accuracy metric stopped improving (start with 1 × 10^−3^ reduce factor = 0.5).

The code source designed for the pathology classification was deposited in the open-access GitHub resource and is currently available at the following link: https://github.com/Denis21800/Cancer-Patholology-classification-4-channels-1D-Resnet- (accessed on 5 October 2021)

### 2.5. 1D-Residual CNN Model

The next model tested in the framework of this study was built according to the 3D convolution architecture, which is often used to classify a sequence of images, such as video signals (where multiple image frames are concatenated across a temporal dimension to provide a 3D spatial input), medical image slices (magnetic resonance imaging; MRI), etc. The kernel shape for a 3D convolution is specified along three dimensions: depth, height, and width. When considering the convolution operation in terms of a kernel sliding across a multidimensional input array, in a 3D convolution, the kernel slides in three directions. At every step, the dot product is calculated, which provides a 3D output as well.

The input data for this model were a sequence of spectrum images. A distinctive feature of this network is that the model was trained simultaneously based on the data of the spectrum of proteins and metabolites, that is, the input parameters of this model can be both the spectrum of proteins and the spectrum of metabolites, and at the output layer of the model, the probability of the spectrum belonging to one or another class of pathology is determined.

**Figure 3:**
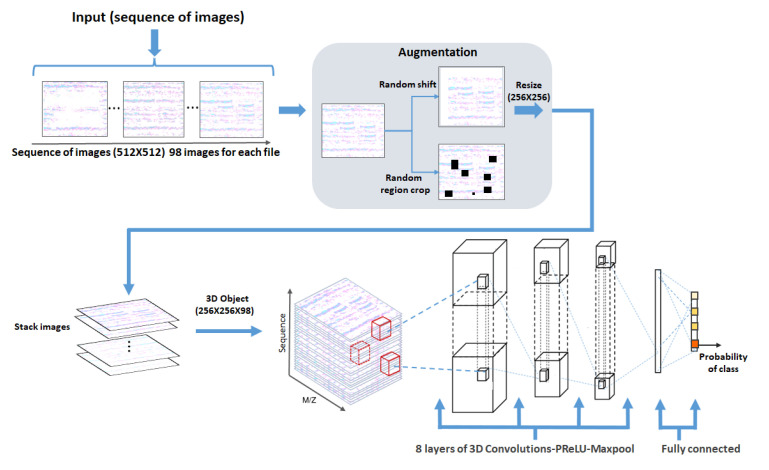
The schematic representation of 3D residual CNN architecture.

Before being fed to the network input, the sequence of images was subjected to additional augmentation: for 30% of the images, one of the randomly selected transformations was applied (random shift of image elements vertically and horizontally, random zeroing of image elements, random crop), and each image in the sequence was reduced to a size of 256 × 256 pixels after augmentation; a 3D object of 256 × 256 × 98 pixels was formed from the sequence of images, which was fed to the input of the neural network (Figure 3).

The 3D convolution model consists of a sequence of eight 3D convolution layers with sub-sampling layers (maxpool3D). The convolution layers are connected in series with two output dense layers. After each convolution layer, a batch normalization layer was applied. The parametric rectified linear units (PReLU) function was used as an activation function (Figure 3 and Table 3).

An Adam optimizer with a reduced learning rate was used when the accuracy metric stopped improving (start with 1 × 10^−3^ reduce factor equal to 0.5). Multiclass cross-entropy was selected as the loss function.

The code source designed for the pathology classification was deposited in the open-access GitHub resource and is currently available at the following link: https://github.com/Denis21800/Pathology-classification_V2.git. (accessed on 5 October 2021).

## 3. Results

### 3.1. Training and Validation of the Model

The model training process lasted for 25 epochs. The models were checked using test and validation datasets. During the learning process, the learning rate parameter was changed for each model. The parameter value decreased when the accuracy of the test set stopped improving. Below are the main indicators of the model training process and the comparative plots of the loss function for the validation dataset (Table 4).

**Figure 4:**
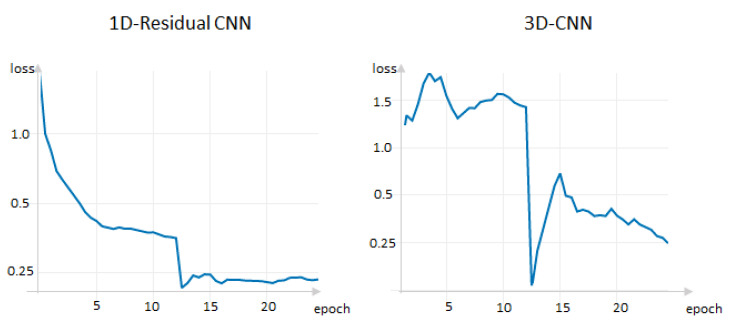
Training curves of two CNN models for average loss curve. The OY axis plots cross-entropy loss function, which is an arbitrary value and depends on the predictive probability of the model.

The average loss curves for the two CNN models used to classify the phenotypes studied and the value of the loss function varies with the learning epoch, which constitutes the loss curve (Figure 4). The loss function curves approach 0 in the region of epochs 12-13 and subsequently stabilize, demonstrating that the model is not overfitted, but the 1D-CNN model shows more stable and smoother convergence during training (Figure 4).

The cross-entropy loss function can be estimated according to the following equation:

$$-\overset{M}{\underset{c=1}{\sum }}y_{o,\;c}\log\left(p_{o,\;c}\right)$$

where “*M*” is the number of classes under consideration (four classes in our case: *CNT*, *OVC*, *PRC*, and *RNC*); *log* is the natural logarithm; “*y*” is a binary indicator (0 or 1) if class label ‘*c*’ is the correct classification for the observation; and ‘*p*’ is the predicted probability observation of class ‘*c*’.

We provided a comparative analysis of the performance of the models based on the learning outcomes and evaluated the score metrics for the two deep learning classifiers and the four traditional machine learning classifiers (Table 5).

As can be seen in Table 5, the 3D-CNN model is characterized by the highest rates of accuracy and response. The model requires much more time for training and preparation of input data, which is approximately 10 times more processing power. Significant time costs are primarily associated with the need to process a large number of image files to allow the graphical presentation of mass spectrometric data. In the future, this problem could be solved by using additional storage optimization tools, using caching packages (e.g., in-memory cache). However, such an optimization was not implemented within the framework of this study.

### 3.2. Distance Matrices

The distance matrix reflects the extent to which the studied phenotypes are based on the analysis of proteomic and metabolomic analysis data using machine learning tools. As can be seen from the results, the mutual distance between the studied pathological conditions and the phenotype of a healthy person is perfectly matched to the expectation, and both models predicted similar results. The control was the most distant from the oncological conditions, while the female oncopathology of ovarian cancer was significantly removed from male oncopathology of prostate cancer, with values of 3.58 and 4.29 for 1D residual CNN and 3D CNN, respectively. The closest molecular components were gender-mixed and gender-specific oncopathologies with kidney/ovarian cancer exhibiting a value of 1.47 and 1.65 for 1D residual CNN and 3D CNN, respectively. Kidney/prostate cancer was significantly distant from 1D residual CNN with a value of 4.63; however, for 3D CNN, the distance between these phenotypes was less than 2.79.

## 4. Discussion

The analysis of large datasets obtained using high performance liquid chromatography–isotope dilution tandem mass spectrometry (HPLC-MS/MS) for proteins and metabolites is based on algorithms that search for peptides/proteins or metabolites in databases by correlating the mass-charge characteristics of tandem spectra theoretically predicted with a fixed threshold of matching accuracy [33]. At present, researchers have access to several search engines, the search algorithms for which are different, and as a consequence, the results of identification of the same sample are not identical [33,34,35,36]. This variability in the results of identification by search engines is due to the high dynamic range of molecular components of blood, as well as the variety of protein isoforms due to alternative splicing, post-translational modification, and amino acid substitutions, which significantly limits the use and reliability of identification algorithms [37]. In the case of metabolic analysis, a significant limitation is the high degeneracy of isobaric compounds as well as the pronounced influence of the epigenetic factors of components of non-endogenous origin, supplied with food, cosmetics, drugs, and waste products of microflora. Therefore, to increase the number and accuracy of identifications in omics studies, there have been a number of studies proposing the use of several identification algorithms for the same “omics” data in order to increase the number of technical and biological repetitions [33,34]. Although these approaches increase the number of identifications by 10–15%, they also significantly increase the cost of research.

Even complex identification of proteins/metabolites does not solve the problem of identification. Most of the obtained tandem spectra remain unidentified, that is, the “dark” proteome and metabolome [9]. Despite the fact that the “dark” proteome/metabolome contains noise signals, the presence of a large volume (up to 70%) of unidentified MS signals indicates algorithmic identification limitations, which are almost impossible to overcome by FDR (false discovery rate) alone, as it leads to a significant distortion of the final results and a decrease in the statistical reliability.

In this regard, we propose a new approach to the analysis of multidimensional “omics” data, both proteomic and metabolomic, for the classification of oncological phenotypes. In this study, we proposed two models of neural networks, 1D residual CNN and 3D CNN, which are based on different convolutional algorithms. The first model presents the MS spectrum in a time-based form, in which the time of the release of the molecular component from the chromatographic column serves as the time component. In the second model, the tandem MS spectrum was transformed into a set of images within a certain time period. As expected, the performance of the 3D-CNN model was 10 times lower than that of the 1D residual CNN (Table 4).

The main difference in the architecture of the two models used is their sensitivity to the data structure. Initially, training of the 1D-CNN model focused on the input of two datasets of the same type (gas chromatography–mass spectrometric data stream with the properties of continuity in retention time (not used at the input) and intensity and values of mass-to-charge ratios), but different from each other in structure (proteomic data flow and metabolomic data flow). The presence of four input data channels determines the selectivity of the model to the structure of the input data. On one hand, this can be regarded as a limitation of the application of the 1D-CNN model in the architectural form as presented in this work, as it is far from possible to obtain proteomic and metabolomic data from a single experiment under laboratory conditions. On the other hand, the presence of two types of structured data can be used to control the correctness of the phenotype classification process. An additional limitation of this model may also be the possible interference of the input values on the *m*/*z* channel between proteomic and metabolomic data, especially in the region up to 300–400 *m*/*z*. However, the risk of such an outcome can be overcome by limiting the scan range to the 400 *m*/*z* value in the proteomic data structure.

From the above information, it can be considered that the classification model with the 3D CNN architecture is free from these limitations, as metabolomic and proteomic data are fed simultaneously at the input. The algorithm to convert these spectra and present them into a three-dimensional image with specified measurement boundaries allows the researcher to ignore the data structure itself. The ability to carry out classification not based on the stream of numerical values, but rather on the analysis of images (converted stream of numerical values), as a surrogate of the original data, allows for a higher accuracy of data classification (Table 5). The reason for the higher accuracy and responsiveness is the generalization and coarsening of the data as it is converted to a graphical form.

However, a few limitations can be specified for the use of the 3D-CNN model as well. In addition to a lower performance in the order of magnitude due to the need to analyze a large array of graphical data, an additional stage appears in the classification algorithm itself, in which data are converted from a stream of numerical values into a graphical form. Unlike 3D CNN, 1D CNN does not require additional data manipulation, which allows it to be used without the risk of loss or distortion. In the case of the 3D-CNN model, the risk of displacement pixilation, replacement, and additional alignment can significantly affect the final result. However, this does not make the model less useful for the analysis of phenotypes. The 3D-CNN classification model seems to be more universal in its application because it does not depend on the data structure and utilizes the converted form of the original data.

The performance indicators of both models were accuracy and recall (at 0.95), which were achieved during trials and data testing (Table 5). A direct indicator of the higher accuracy of the 3D-CNN model was the distance between the classes (Figure 4). In the 3D-CNN model, the distances between the gender-specific OVR and PRC classes and the control group were equally removed, while the distance between the PRC and the 1D-CNN model was significantly lower (Figure 4). Simultaneously, the distance between the control group and the RNC (gender-independent phenotype) was the same in both of the models. Thus, the 3D-CNN model is more sensitive, owing to its more efficient algorithm for the classification and analysis of graphic images rather than the mixing of the control group by gender.

An important difference between machine learning methods is the ability to work with raw files converted to the standard ‘mgf’ format, which is the same for all HPLC-MS/MS platforms, ensuring the versatility of the proposed approaches in the future.

In addition, in our study, we proposed a new approach for the development of network medicine and the study of human diseases. The phenotype of cancer is not a consequence of a violation of the expression level or the presence of mutation (s) in one effector gene, but reflects the spectrum of genetic and epigenetic events [38]. The similarity of pathological processes accompanying the development of oncological diseases and the interconnected nature of interactions of molecular components makes it possible to map the distance between pathophenotypes by analyzing cellular and circulating molecular components. For the three oncophenotypes and the phenotype of a healthy person, we built a distance matrix to determine their similarity.

Introducing the obtained results in clinical settings is difficult. Mass spectrometry is still an advanced instrumentation for the purpose of research. It is necessary to scale-up such research on significantly larger cohorts. The small dimensionality of the study cohort allows us to make some intermediate conclusions. 

Unlike traditional proteomics characterization approaches, we utilized machine learning for the complete mass spectrometric dataset, which is a part of identifiable data and a fraction of unassigned data. Such an approach can classify not merely control from disease, but can also distinguish closely related phenotypes and separate gender-specific phenotypes. At least, that could be useful to support decision making when traditional diagnostics is hard or ambiguous. In addition, if raw mass spectrometry data are not available, the 3D model can operate with images of mass spectrometry data. Certainly, it takes a longer time to analyze and collect multiple images instead of the analysis of a data stream, but expands 3D model properties to multipurpose and more general applications in medicine.

## 5. Conclusions

**Figure 5:**
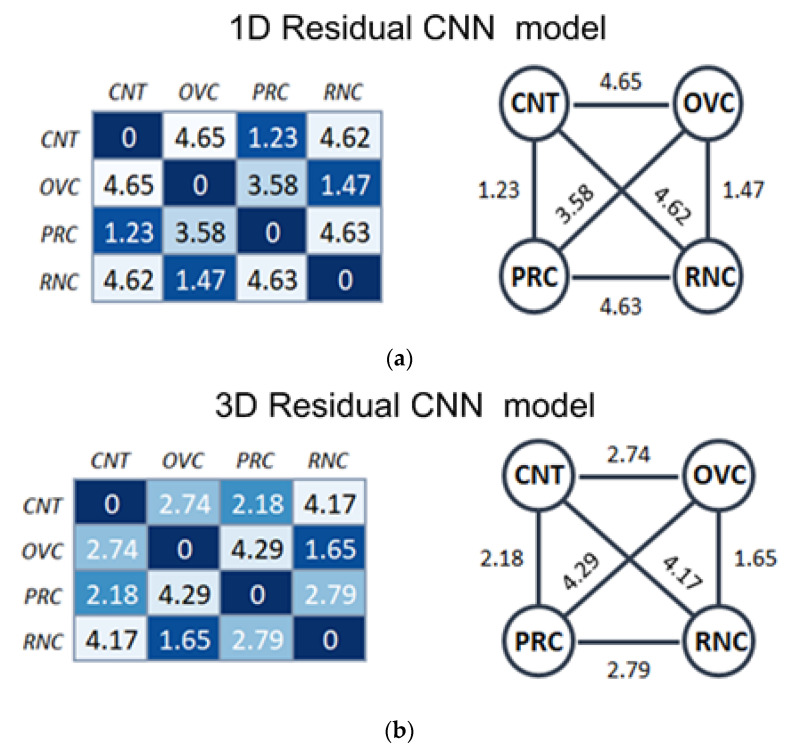
Confusion matrix of neural network predictive results obtained on the testing dataset for Table 1. One-dimensional residual CNN (**a**) and 3D CNN (**b**). The number of correctly recognized files is indicated in the interception of certain pathology unless the number is in the intercept between different phenotypes. CNT—control, OVC—ovarian cancer, RNC—kidney cancer, PRC—prostate cancer.

In this study, we carried out a comparative analysis of the efficiency of classification of the same input data using two neural networks with different architectures. Despite the presence of phenotypes that are similar in etiology and pathogenesis, and different in terms of gender, both 1D and 3D neural networks successfully classified the studied oncological phenotypes with a recall level of more than 0.78, taking into account augmentation (Table 5). The results of the classification of proteomic-metabolomic data showed significant differences between the studied phenotypes. As can be seen in the graphs, for both neural networks (Figure 5), the pathological conditions were significantly removed from the phenotype of a healthy volunteer. The gender-specific oncopathologies were also distant from each other.

The classification accuracy of the 3D-CNN model is slightly higher than that of the 1D-CNN model, which is due to the possibility of generalizing structurally different (metabolomic and proteomic) data at the entrance to the system, owing to their conversion into a graphical form of data. However, this also determines the need for higher computing power and data analysis time. In addition, unlike the 1D CNN, the 3D CNN requires a preliminary conversion of numerical data arrays into a graphic format. The main advantage of this model architecture is the lack of selectivity to the data structure, which avoids the simultaneous presence of metabolomic and proteomic data for classification, and is capable of efficient classification in the presence of one or two streams of input data.

The neural networks proposed in our study were adapted for any HPLC-MS/MS platform. In this case, the HPLC-MS/MS spectrum can be presented in the form of a time base, the parameters of which include the time of release of the molecular component from the chromatographic column (time component), as well as the mass-charge characteristic and signal intensity, depending on the concentration of the molecular component. In addition, the mass spectrum in the time base sweep can be represented as a heat map (3D convolutional model), the axes of which are the retention time (RT) and the mass-to-charge ratio (*m*/*z*), while the color of the pixel reflects the signal intensity.

## Figures and Tables

**Table 1:** Sizes of datasets.

Dataset	Training Size	Testing Size	Validating Size
Original	217	120	–
Augmented	1302	420	300
Original	203	120	–
Augmented	1302	420	300

**Table 2:** 1D-residual CNN model parameters.

Layer Name	Kernel Size, Filters	Number of Blocks	Stride
Conv1	(32, 64)	1	2
Conv2_x	(7, 64)	4	2
	(7, 64)		
Conv3_x	(7, 128)	3	2
	(7, 128)		
Conv4_x	(7, 256)	3	2
	(7, 256)		
Conv5_x	(7, 512)	4	2
	(7, 512)		
Avgpool, kernel size = 3			
Dense (512 × 4)			

**Table 3:** 3D-CNN model parameters.

Layer Name	Kernel Size, Filters	Stride
Conv1	((7, 9, 9), 16)	(1, 1, 1)
Maxpool1	(3, 3, 3)	(1, 1, 1)
Conv	((5, 7, 7), 16)	(1, 1, 1)
Maxpool2	(2, 2, 2)	(1, 1, 1)
Conv3	((5, 7, 7), 32)	(1, 1, 1)
Maxpool3	(2, 2, 2)	(1, 1, 1)
Conv4	((2, 5, 5), 32)	(1, 1, 1)
Conv5	((2, 5, 5), 64)	(1, 1, 1)
Conv6	((1, 3, 3), 128)	(1, 1, 1)
Conv7	((1, 3, 3), 256)	(1, 1, 1)
Conv8	((1, 3, 3), 512)	(1, 1, 1)
Dense (4096, 64)		
Dense (64 × 4)		

**Table 4:** Indicators of the model learning process.

CNN	Epochs	Total Training Time (GPU NVidia GTX-1650)	Learning Rate
1D residual CNN	25	23 min	1 × 10^−3^ → (reduced to) → > 1.25 × 10^−4^
3D residual CNN	25	145 min	1 × 10^−3^ → (reduced to) →5 × 10^−5^

**Table 5:** Training and evaluation metrics of the CNN models.

CNN	Input	Dataset	Accuracy	Recall	F1-Score
1D-residual CNN	4 channels of raw data: *m*/*z* (proteomics) Intensity (proteomics) *m*/*z* (metabolomics) Intensity (metabolomics)	Train	0.953	0.941	0.956
		Test	0.812	0.796	0.801
		Validation	0.784	0.781	0.781
	Class	Control	0.69	0.86	0.76
		Ovarian cancer	0.79	0.95	0.85
		Prostate cancer	0.89	0.77	0.82
		Kidney cancer	0.92	0.7	0.79
3D CNN	Sequence of spectrum images Proteomics spectrum or metabolomics spectrum	Train	0.974	0.968	0.972
		Test	0.893	0.889	0.893
		Validation	0.861	0.850	0.854
	Class	Control	0.79	0.95	0.86
		Ovarian cancer	0.83	0.95	0.88
		Prostate cancer	0.94	0.78	0.85
		Kidney cancer	0.91	0.83	0.86

## Data Availability

The code source designed for the pathology classification was deposited in the open-access GitHub resource and is currently available at the following link: https://github.com/Denis21800/Cancer-Patholology-classification-4-channels-1D-Resnet- (accessed on 5 October 2021) The code source designed for the pathology classification was deposited in the open-access GitHub resource and is currently available at the following link: https://github.com/Denis21800/Pathology-classification_V2.git (accessed on 5 October 2021). Proteomic and metabolic data as ‘mgf’ peak list file were deposited in the Mendeley Repository. Datasets of the control group and prostate cancer for machine learning are available under the following link: Malsagova, Kristina (2021), “Unassigned Mass Spectrometry Data for Machine Learning”, Mendeley Data, V1, doi:10.17632/ycw25mpjb6.1. Datasets of ovarian cancer, renal cancer and prostate cancer are available under the following link: Kaysheva, Anna; Stepanov, Alexander; Petrovsky, Denis (2021), “Machine Learning of MS dataset”, Mendeley Data, V2, doi: 10.17632/2rcc8488hx.2.
